# Prevalence and Annual Health Insurance Cost of Endometriosis in Hungary—A Nationwide Study Based on Routinely Collected, Real-World Health Insurance Claims Data

**DOI:** 10.3390/healthcare11101448

**Published:** 2023-05-16

**Authors:** Tímea Csákvári, Dalma Pónusz-Kovács, Luca Fanni Kajos, Diána Elmer, Róbert Pónusz, Bettina Kovács, Ákos Várnagy, Kálmán Kovács, József Bódis, Imre Boncz

**Affiliations:** 1 Institute of Health Insurance, Faculty of Health Sciences, University of Pécs, 7621 Pécs, Hungary; 2 National Laboratory on Human Reproduction, University of Pécs, 7622 Pécs, Hungary; 3 Department of Obstetrics and Gynecology, Clinical Center, Medical School, University of Pécs, 7622 Pécs, Hungary

**Keywords:** endometriosis, cost, prevalence, epidemiology, Hungary

## Abstract

Endometriosis is a disease that is often diagnosed late and that may lead to significant reduction in quality of life and serious complications (e.g., infertility). We aimed to assess the prevalence and the annual, nationwide health insurance treatment cost of endometriosis in Hungary using a quantitative, descriptive, cross-sectional method, focusing on the year 2019. We used claims data obtained from the Hungarian National Health Insurance Fund Administration (NHIFA). Patient numbers, total and age-specific prevalence, annual health insurance expenditure, and the distribution of costs across age groups were determined. The NHIFA spent a total of HUF 619.95 million (EUR 1.91 million) on endometriosis treatment. The highest number of patients and prevalence (10,058 women, 197.3 per 100,000) were found in outpatient care. In acute inpatient care, prevalence was substantially lower (23.5 per 100,000). Endometriosis, regardless of its type, affects 30–39-year-olds in the highest number: 4397 women (694.96 per 100,000) in this age group were affected in 2019. The average annual health insurance expenditure per capita was EUR 189.45. In addition to early detection and diagnosis of endometriosis, it is of pivotal importance to provide adequate therapy to reduce costs and reduce the burden on the care system.

## 1. Introduction

Endometriosis is a chronic, estrogen-dependent gynecological disease that is associated with severe pelvic pain and infertility and increases the risk of developing other malignant lesions, as well as immunological and atopic diseases [1,2,3,4]. In addition to the causes of the development and spread of this disease, with its varied clinical presentation, its full impact on the body and its possible manifestations outside the reproductive tract are still not fully understood. Diagnosing and treating this possibly systemic and multidisciplinary disease can therefore be challenging [5] and can take years [6]. The average time from first symptoms to diagnosis among Canadian women was estimated to be approximately 5.4 years [7]. According to another study, more than 50% of US patients waited longer than 6 years for a diagnosis [8]. In Europe, an average wait of 10.4 years was reported among women in Austria and Germany, where 74% of women were misdiagnosed at least once [9]. In Hungary, the average diagnostic delay was 2.01–3.9 years [10,11].

Many factors can contribute to diagnostic delays [12], such as the lack of reliable non-invasive tests, misleading symptoms, and late arrival to care centers with the appropriate diagnostical tools—of which laparoscopic surgery is considered to be the most correct form [5,13]. The stage of the disease must also be taken into consideration when choosing the best treatment method. The classification system of the American Society of Reproductive Medicine (ASRM) distinguishes four stages of endometriosis. In Stage I (minimal) and Stage II (mild), deep lesions and thin adhesions are absent or rare, while in Stage III (moderate) and Stage IV (severe), larger adhesions and endometrioma can occur and possibly spread to other organs, further decreasing the patient’s quality of life [14].

Considering the above facts, the prevalence of endometriosis in women of reproductive age ranges widely and is difficult to determine. A recent study estimates the prevalence of the disease at 18% globally, 17% in Europe, 19% in the Americas, 26% in Africa, and 36% in Asia [15]. Regarding its incidence, most new cases are found among 25–45-year-olds [16,17,18,19].

Diagnostic delay can have a negative impact not only on quality of life and thus productivity [20,21,22,23,24], but can also increase the cost of treatment. A US study with 11,793 participants set the average diagnostic delay to 763.9 days and the total cost per capita related to endometriosis to USD 3553. Among women who took the longest to be diagnosed (1505.9 days), this was USD 4794, while among those who took the shortest time (90.2 days), it was less than half (USD 2082). The greatest cost drivers were ambulatory care (59.1%) and pharmaceutical reimbursement (17.7%) [25].

Studies measuring the burden of the disease and quality of life associated with endometriosis among Hungarian women are scarce, and their results vary [10,11,26,27].

### The Hungarian Health Care and Health Insurance System

The Hungarian compulsory health insurance system is based on a single payer (National Health Insurance Fund Administration, NHIFA) which covers 98–99% of the population. Any healthcare provider, regardless of ownership (public, non-profit, church, private, etc.), can initiate a financing contract with the NHIFA. These providers send monthly data on the volume of care and services covered by the contract and the patient population served, which forms the basis for reimbursement.

It is mandatory to be a member of the health insurance risk pool in Hungary. The insured will be entitled to health insurance benefits by right if they pay contributions (a percentage of their monthly income) to the NHIFA, while those without income (pensioners, schoolchildren, etc.) are insured by contributions from the central budget. The role of private health insurance companies is supplementary and covers mainly amenities (single room, LCD television, etc.). Only a small proportion of the Hungarian population has private health insurance.

The regulation and functioning of the Hungarian health care system and its structural characteristics have been described in detail elsewhere [28,29].

We aimed to assess the prevalence and the annual health insurance treatment cost of endometriosis in Hungary.

## 2. Materials and Methods

The epidemiological and health insurance disease burden of different types of endometrioses were analyzed using a quantitative, descriptive, cross-sectional method in Hungary, focusing on the year 2019.

The research used a nationwide database of real-world data from the sole publicly funded health insurance organization of Hungary, the National Health Insurance Fund Administration (NHIFA) [30], as it contains claims data for all Hungarian, publicly funded healthcare providers. We identified endometriosis according to the following ICD codes (10th Revision): endometriosis of the uterus (N80.0), ovary (N80.1), fallopian tube (N80.2), pelvic peritoneum (N80.3), rectovaginal septum, and vagina (N80.4), as well as other (N80.8) and unspecified endometriosis (N80.9).

The utilization and reimbursement of 14 treatment types were assessed: general practitioner care; home (nursing) care; care in care centers; outpatient care; acute and chronic inpatient care; medical imaging (CT, MRI); laboratory diagnostics; pharmaceuticals; medical aids; disposable instruments, implantations and medicaments falling under itemized accounts; ambulance service; patient transportation. (For ambulance service, data on patient numbers were available and thus analyzed only.) Regarding acute and chronic inpatient care, cases reported with a primary diagnosis of endometriosis were evaluated only.

To determine the health insurance burden—from the health insurer’s viewpoint—of the diseases included in the study, annual health insurance expenditure and the distribution of costs by age group were determined. Expenditures are given in EUR (at the average EUR to HUF exchange rate from 2019).

For epidemiological analysis, we also examined the annual patient and case numbers, as well as the total and age-specific prevalence per 100,000 female population. To calculate the prevalence, we obtained age-specific data from the Hungarian Central Statistical Office for the female population in 2019.

We also estimated the average length of stay as the ratio of the number of patients to the number of days of care provided in acute inpatient care. To avoid duplication between different treatment types and to ensure comparability between different ICD codes, the number of patients receiving outpatient care was selected to calculate the prevalence for all disease subgroups. The annual case numbers per patient were also determined to further analyze the utilization of each care type. For that, we divided the case numbers of all 14 care types assessed with the corresponding patient numbers for all ICD codes.

## 3. Results

### 3.1. Epidemiology

In Hungary, the highest number of patients (10,058 women) was found in outpatient care in 2019. A total of 8174 women consulted their general practitioner about some form of endometriosis, and 4372 women used reimbursed pharmaceuticals (Table 1).

Out of the different disease types assessed, the largest ratio belonged to unspecified endometriosis (N80.9) in outpatient care (67.88%). Ovarian endometriosis also occurred in a significant proportion (15.99%), while all the other types accounted for 16.14%. In addition, almost half (39.12%) of the acute inpatient population was found to have ovarian endometriosis.

The mean age across the three types of care with the highest patient numbers was roughly the same: outpatients average 37.5 years, those receiving reimbursed medication 38.8 years, and women in general practice 38.4 years. Patients attending laboratory diagnostics and acute inpatient care tended to be younger (36.6 and 35.8 years, respectively).

**Figure 1:**
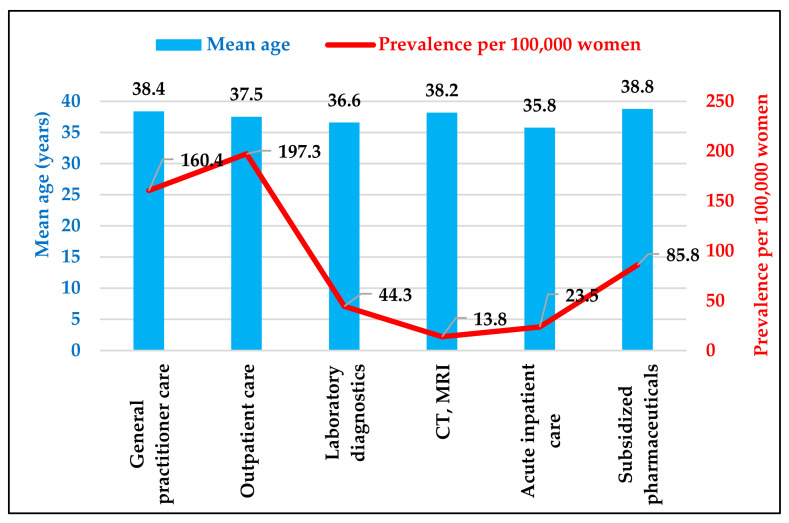
Average age and prevalence of women with endometriosis by different types of care. Source: NHIFA, 2019.

The prevalence defined for the different types of care varied considerably. The highest prevalence was observed in outpatient care (197.3 per 100,000 women), followed by the prevalence based on general practice (160.4 per 100,000 women), while in acute inpatient care it was substantially lower (23.5 per 100,000 women), and the prevalence based on the number of patients using prescribed medicaments was about half that calculated based on general practitioner care (85.8 per 100,000 women) (Figure 1).

**Figure 2:**
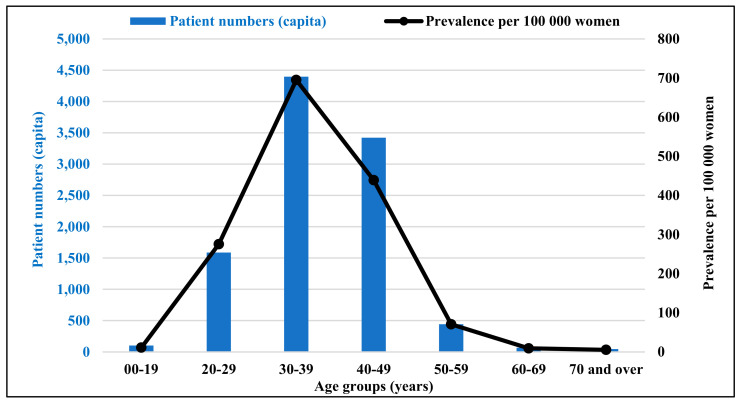
The number of patients in outpatient care and prevalence per 100,000 women of each age group. Source: NHIFA, 2019.

Figure 2 shows the annual outpatient numbers and the prevalence per 100,000 women of each age group. Endometriosis, regardless of its type, affected 30–39-year-olds in the highest number: 4397 women (694.96 per 100,000) in 2019. This was followed by the 40–49 age group (3422 patients, 438.94 per 100,000), and then the 20–29 age group (1586 patients, 275.27 per 100,000). For two disease types, we observed a higher proportion of women aged 40–49 years: endometriosis of the uterus (40.71%) and endometriosis of the fallopian tube (44.26%). The rates for women aged 20–29 years were highest for ovarian (17.04%) and peritoneal endometriosis (16.28%).

### 3.2. Health Insurance Costs

The National Health Insurance Fund Administration of Hungary spent a total of HUF 619.95 million (EUR 1.91 million) on the treatment of endometriosis in 2019. The exact health insurance expenditure for each type of care is shown in Table 2. The largest share of expenditure was for the reimbursement of pharmaceuticals (48.20%), acute inpatient care (31.63%), and outpatient care (6.25%).

Out of the different subgroups analyzed, the largest expenditure share and patient numbers belonged to unspecified cases (67.07%; EUR 1.28 million), followed by ovarian endometriosis (21.47%; EUR 409,160). Every other form added up to 11.46% of total expenditure in 2019 (Table 2).

For the relevant cost drivers, we examined the differences in the distribution of expenditure between the different anatomical locations. The share of pharmaceutical reimbursement for each form of endometriosis ranged from 8.58% (fallopian tube) to 62.28% (unspecified), whereas the treatment of the ovary (70.70%) and fallopian tube (63.32%) was associated more with acute inpatient care. The lowest proportion of inpatient care costs was observed for the rectovaginal septum and vagina (11.69%) and the unspecified cases (17.07%). Apart from the latter, the costs associated with general practitioner care ranked third for each of these conditions (fallopian tubes: 5.08%; uterus: 13.53%). It is also worth highlighting the distribution of costs for rectovaginal septum and vaginal endometriosis, where the proportion of chronic inpatient care (10.78%) was much higher than for the other ICD codes (elsewhere, this type of care accounted for an average of only 1.38%).

**Figure 3:**
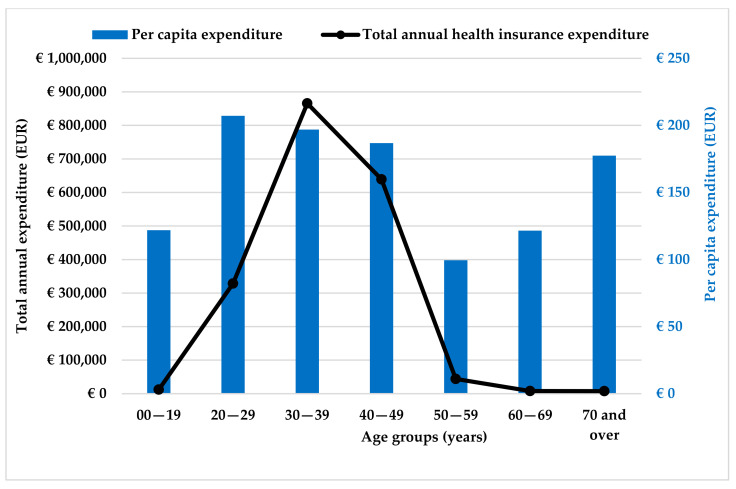
Total annual health insurance expenditure and average annual expenditure per patient by age group (in EUR). Source: NHIFA, 2019.

The total and per capita expenditures of different age groups are shown in Figure 3. The highest expenditure belongs to those aged 30–39 years (45.44%; EUR 865,780). For the treatment of 40–49-year-olds, EUR 639,110 (33.54%) was reimbursed, while for the 20–29 age group, EUR 328,500 (7.24%) was reimbursed by the NHIFA. In contrast to the total expenditure, the 40–49-year-olds demonstrated the highest expenditure for endometriosis of the uterus (47.40%) and fallopian tubes (42.91%).

Based on the data for outpatient care, we also calculated the average annual health insurance expenditure per capita, which averaged EUR 189.45 in 2019. The lowest per capita health insurance expenditure was for treating endometriosis of the rectovaginal septum and vagina (EUR 114.58), while the highest was for treating ovarian endometriosis (EUR 254.45), but the expenditure for treatment of the pelvic peritoneum (EUR 192.25) was also significant compared to the other types of diseases studied. This indicator was much higher, however, for hospitalized patients, where the annual cost per patient was EUR 502.74 in 2019.

The average length of stay when endometriosis was the main diagnosis in acute inpatient care was 2.8 days. The lowest length of stay recorded was for patients with endometriosis of the rectovaginal septum and vagina (1.6 days), while the longest was for endometriosis of the uterus (5.33 days).

Correlation analysis was used to examine the relationship between per capita expenditure (expenditure for a given type of care divided by the number of patients recorded for each ICD code) and average age (also for a given type for each ICD code). The detailed results can be seen in Table 3.

We found a negative relationship between laboratory diagnostics (*r* = −0.503; *p* = 0.020) and subsidized medicaments (*r* = −0.356; *p* = 0.097), although the latter was not statistically significant. These results mean that with younger age comes a higher per capita cost among endometriosis patients.

However, we found a moderate, positive, although not significant, relationship between the per capita cost of acute inpatient specialist care (*r* = 0.405; *p* = 0.060) and average age. In conclusion, acute inpatient care, including treatments requiring higher expenditure, tends to affect older patients in NHIFA-funded hospitals.

We also examined the existence of relationships between age and the annual number of cases per patient. For patient transportation (*r* = 0.649; *p* = 0.002), ambulatory service care (*r* = 0.879; *p* < 0.001), home care (*r* = 0.981; *p* < 0.001), and chronic inpatient specialty care (*r* = 0.995; *p* < 0.001), older women typically present more than once a year. In contrast, an inverse relationship was found for outpatient care (*r* = −0.453; *p* = 0.036), where women of younger ages return more than once a year more so than older patients.

Additionally, we also detected a statistically significant, strong, and positive association between mean age and mean length of stay in acute inpatient care, suggesting that older patients typically require longer hospital stays (*r* = 0.877; *p* < 0.001).

## 4. Discussion

Our analysis aimed to determine the epidemiological and annual health insurance cost of endometriosis in Hungary; therefore, we performed our analysis on a nationwide dataset covering the whole Hungarian population covered by the only health care financing agency in a single-payer system. We found that the prevalence of endometriosis is 275.27 per 100,000 among 20–29-year-olds, 694.96 per 100,000 in 30–39-year-olds, and 438.94 per 100,000 in 40–49-year-olds, based on outpatient data. Our results are in line with those based on real-life data from France (0.90% for 15–49-year-olds) [31].

Epidemiologic studies assessing endometriosis show different levels of prevalence, with a large variance influenced by the number of patients and their methodological specificities. In a literature review and meta-analysis of 69 studies, the prevalence of endometriosis was estimated to be between 0.2% and 71.4%, with prevalence typically lower in population-based analyses with the largest number of items (2.4%), and 4.3% in national/regional questionnaire surveys (10,000 population: 2.4%, over 10,000 population: 4.4%), while the highest prevalence (and variance) was found in studies that took place in a hospital/clinical setting (15.9%) [32].

In addition to outpatient specialist care, a significant proportion of Hungarian women also visited their general practitioners about their illnesses, and a large proportion of them also received subsidized medications—the latter being the area with the highest health insurance expenditure. Although general practitioners have a gatekeeping role in Hungary, specialized gynecological services available at the outpatient level can be used without a GP referral. Our results show that more women go straight to specialist care with their symptoms. The use of specialist care as the first place where a patient presents with a problem may also be explained by the fact that women tend to seek help only for more serious complaints and pain. The direct and indirect costs associated with the care of endometriosis can be a significant burden for individuals and society [33]. In our analysis, we found the cost per patient of treating endometriosis to have been EUR 189.45 in 2019, based on the outpatient specialty care element number. In contrast, the cost per capita of hospital care is already higher, at EUR 502.74. This is still well below the average per capita expenditure found in the literature. For instance, a study on 10 countries found the per capita expenditure to be EUR 9579 in 2008, of which indirect costs linked to productivity loss produced the largest proportion (66%, EUR 6298), whereas direct costs amounted to EUR 3281 per capita [34]. Annual per capita expenditure was EUR 8768 in Sweden in 2010 (based on 2019 prices), of which direct costs amounted to EUR 4282 and indirect expenditure was EUR 4486 [35]. In Austria, the average annual cost per person was EUR 7712 in 2011, with 73% of this being direct cost [36]. In Germany, for example, the direct cost of treatment is USD 4846.99, in Canada it is USD 1109.45, and in the US it is up to ten to fifteen times higher (up to USD 16,574, according to Gao et al.). There are several possible reasons for this phenomenon. For example, there were significant differences between the income levels of doctors in Hungary and in other countries (in 2019), which were then reduced by a reform of the Hungarian health care and human resources system [37]. Due to the different natures of the health insurance systems, administrative costs can also be significantly higher in countries such as the US, where private insurers dominate. Differences in the pharmaceutical market (e.g., price regulation) can also lead to significant differences in health expenditure on endometriosis between countries.

According to the available studies on expenditure, the direct public expenditure per capita for endometriosis—as we have also assessed—can be significant, with differences of up to ten times between countries. However, the methodologies used to determine the costs show at least as much variation, meaning that the data reported must be treated with caution. One should keep in mind that the health systems of the countries also differ (e.g., the USA, where the annual per capita cost in the year following diagnosis is USD 13,199, which is equivalent to EUR 9046.8 at the exchange rate in the year of the study) [38]. In addition, the indirect costs often tend to be even higher. Although we have not been able to measure them, we can assume that there may be significant indirect costs associated with endometriosis in Hungary [33].

In general, we have also observed that in most publications, acute inpatient care accounts for the largest share of direct costs [38,39], whereas we have identified pharmaceutical reimbursement as the primary cost driver. The discrepancy may be due to methodological differences in international studies (no drug costs were examined), but it may also be due to differences in care protocols.

In the US, the average length of acute inpatient stay in the year following diagnosis was found to be 1.6 days [39], while another study found that it was 2.6 days in 2014–2015 [40]. In the national context, similar values were obtained (2.8 days (1.6–5.33)).

Despite the relatively low number of patients, acute inpatient care still involved a significant amount of health insurance expenditure in our study. To reduce costs, it may be important to support innovative, more cost-effective therapies, or those that can be delivered at lower levels of care, for those conditions for which higher than average inpatient admission rates have been found. For example, there have been significant technological advances in medical imaging technologies such as transvaginal ultrasound or MRI in recent years, which is why Chapron et al. suggest that exploratory laparoscopy is no longer always justified for the diagnosis of endometriotic lesions. The authors base the diagnosis on a structured process combining patient interviews, clinical examination, and imaging [41].

We found a negative relationship between laboratory care (*r* = −0.503; *p* = 0.020) and pharmaceutical reimbursement (*r* = −0.356; *p* = 0.097). This means that the younger age group incur a higher per capita cost. In conclusion, efforts should be made on the part of both the patient and the care provider to diagnose the disease as early as possible in life. In addition to early detection and diagnosis of endometriosis, it is of pivotal importance to provide therapy at the lowest possible level of care to reduce costs and reduce the burden on the care system, in terms of allocative effectiveness. One study showed that 76.8% of general practitioners were visited by women with typical symptoms of endometriosis, but only 36% of them diagnosed them with endometriosis when confronted with these symptoms. Thus, in addition to patient education, the early recognition of symptoms should also be emphasized in the training of health professionals. Our analysis shows that Hungarian women visit their general practitioners in high numbers, with the second highest prevalence in Hungary being found in this type of care [42].

We also found that older endometriosis patients typically use patient transportation, ambulatory care, home care, and chronic inpatient care more often. In contrast, an inverse relationship was found in outpatient care (*r* = −0.453; *p* = 0.036), where younger women return more than once a year more so than older patients. A similar result was found by Grundström et al. in a study of Swedish women, where they found that those under 30 years of age also had a higher rate of use of general practitioner’s care and gynecological specialist services [35]. The likely reason behind this result is the higher level of health literacy, health awareness, and perception of the importance of fertility among younger women. In addition, we also found a significant, strong, positive association between mean age and mean length of stay in active inpatient care. This finding suggests that older female patients typically need to be treated for a longer period (*r* = 0.877; *p* < 0.001), suggesting that older age is associated with a higher risk of surgery and longer recovery times. Therefore, it is also more beneficial for the patient and the health insurer to have these procedures performed at a younger age.

In our research, some levels of care had very low or no utilization at all regarding endometriosis. For example, the use of medical aids, home (nursing) care, and patient transport is not common in the treatment of endometriosis. Among the ICD codes, the high use of the code without a specific name (N80.9) compared to the other codes shows that the coding does not specify the location of endometriosis (uterus, ovary, etc.), especially in cases where it is not essential to record the exact diagnosis for a given treatment (e.g., prescription of painkillers)—which may lead to data bias. The use of an ICD code independent of the localization may also be explained by the need to classify the disease in the appropriate homogeneous disease groups (HDGs) (e.g., ovarian endometriosis, coded N80.1), or even for people with certain diseases to claim the personal income tax benefit, which has been available in Hungary since 2019 [43].

Knowing the severity of endometriosis is also of key importance, as each stage differs and causes various symptoms and clinical manifestations, requiring different treatment plans. The 11th Revision of the ICD already contains much more detailed codes for identifying location and severity than the 10th, and the “Endometriosis Severity Scale Value” has been developed, which allows the disease to be coded separately according to its severity (three categories) [44]. An appropriate coding system that makes it possible to locate and describe severity accurately will greatly facilitate research into the disease and help to identify any unknown influences and risk factors and the likely outcome of treatment [45]. The success of surgical interventions is also greatly enhanced when patients (in the more severe stages) are treated in endometriosis centers or assisted reproduction centers, where the most effective treatment of this disease can be guaranteed.

At the time of this study, there is no valid clinical guideline for the treatment of endometriosis in Hungary. The European Society of Human Reproduction and Embryology (ESHRE) renewed its guidelines in 2022, and it is necessary that their application and incorporation into the Hungarian health care system be promoted [46].

## 5. Conclusions

The strength of our analysis is that we present national endometriosis-related utilization and expenditure data through real-world, routinely collected claims data. Understanding these indicators is key for health policymakers, as we can better understand the burden of each type of care and identify where allocative and technical efficiency needs to be increased. A suitable patient population can be managed at a lower level of care through the introduction and promotion of innovative diagnostic and therapeutic procedures. Modern endometriosis management should be personalized through a patient-centered, multimodal, and interdisciplinary approach.

However, our research also has some limitations. We have not been able to investigate indirect costs, which can impose significant costs on patients and their families (e.g., cost of care, travel, absence from work, etc.). We also did not have data on other socio-demographic characteristics of the patient population that are likely to cause significant differences in individual cost, quality of life, and prevalence between subgroups (such as marital status, number of children, urban-rural location, and education). We do not have data on private health care utilization, neither regarding expenditure nor the indirect costs patients have to pay regarding their illness.

We also could not determine the exact length of diagnostic delay among Hungarian women with endometriosis. However, it was important to describe the problem of delay, even though no such data were available for our research. Delay negatively affects the quality of life of the individual, as it puts a burden on the health care system, especially on the more costly specialist care. For an efficient healthcare system, it is of pivotal importance to diagnose diseases promptly and to treat them at the lowest possible level, if possible. This can be achieved, among other things, through appropriate patient education and access to services and modern, effective/efficient diagnostic tools. The main aim of our analysis was (also) to draw attention to the significant burden of endometriosis, which provides the basis for ensuring the above-mentioned solutions and health policy decisions.

Based on our results, our main conclusion is to call for the adequate diagnosis of endometriosis patients at an earlier stage of life, as timely treatment can improve the quality of life of the individual and reduce the burden on health insurance as well.

## Figures and Tables

**Table 1:** Annual patient numbers according to different types of endometriosis (capita).

Types of Care	Uterus (N80.0)	Ovaries (N80.1)	Fallopian Tube (N80.2)	Pelvic Peritoneum (N80.3)	Rectovaginal Septum, Vagina (N80.4)	Other (N80.8)	Unspecified (N80.9)	Total	Distribution		
									%	95%CI	
General practitioner care	1075	1519	90	307	64	349	4770	8174	30.41	29.41	31.40
Home care	0	0	0	0	0	0	2	2	0.01	0.00	1.20
Patient transportation	19	1	0	0	0	4	4	28	0.10	0.00	1.30
Ambulatory service	10	1	0	0	1	2	9	23	0.09	0,00	1.28
Outpatient care	877	1608	61	258	69	358	6827	10,058	37.42	36.47	38.36
Care in care centers	6	3	0	0	1	0	17	27	0.10	0.00	1.30
Laboratory diagnostics	209	286	14	32	11	69	1639	2260	8.41	7.26	9.55
CT, MRI	59	73	3	29	3	31	507	705	2.62	1.44	3.80
PET	0	0	0	0	0	0	0	0	0.00	0.00	0.00
Acute inpatient care	89	469	10	63	5	57	506	1199	4.46	3.29	5.63
Chronic inpatient care	0	4	0	3	1	1	14	23	0.09	0.00	1.28
Disposable instruments, implantations and medicaments falling under itemized accounts	0	0	0	0	0	0	0	0	0.00	0.00	0.00
Subsidized medicaments	470	423	13	83	29	117	3237	4372	16.26	15.17	17.36
Subsidized medical aids	2	1	0	0	1	3	4	11	0.04	0.00	1.24
Total	--	--	--	--	--	--	--	26,882	100.00		

Source: NHIFA, 2019.

**Table 2:** Annual health insurance treatment cost of endometriosis according to the type of care (EUR).

Type of Care	Uterus (N80.0)	Ovaries (N80.1)	Fallopian Tube (N80.2)	Pelvic Peritoneum (N80.3)	Rectovaginal Septum, Vagina (N80.4)	Other (N80.8)	Unspecified (N80.9)	Total	Distribution		
									%	95%CI	
General practitioner care	14,118	20,788	1185	3813	784	4912	71,236	116,835	6.13	5.99	6.27
Home care	0	0	0	0	0	0	433	433	0.02	0.00	0.16
Patient transportation	703	116	0	0	0	176	754	1748	0.09	0.00	0.23
Ambulatory service	*--*	*--*	*--*	*--*	*--*	*--*	*--*	*--*	*--*	*--*	*--*
Outpatient care	8848	16,478	906	2456	754	3403	86,164	119,009	6.25	6.11	6.38
Care in care centers	124	46	0	0	5	0	314	489	0.03	0.00	0.17
Laboratory diagnostics	1532	2013	52	159	30	487	11,976	16,249	0.85	0.71	0.99
CT, MRI	8544	11,547	468	4319	509	4069	80,984	110,441	5.80	5.66	5.93
PET	0	0	0	0	0	0	0	0	0.00	0.00	0.00
Acute inpatient care	48,013	289,284	5884	25,063	924	15,422	218,196	602,787	31.63	31.52	31.75
Chronic inpatient care	0	2373	0	2475	852	852	11,786	18,338	0.96	0.00	1.10
Disposable instruments, implantations and medicaments falling under itemized accounts	0	0	0	0	0	0	0	0	0.00	0.00	0.00
Subsidized medicaments	22,450	66,491	798	11,317	3625	17,796	795,940	918,417	48.20	48.10	48.30
Subsidized medical aids	37	21	0	0	422	88	162	730	0.04	0.00	0.18
Total	104,368	409,157	9293	49,602	7906	47,205	1,277,945	1,905,476	100.00		
Distribution (%)	5.48	21.47	0.49	2.60	0.41	2.48	67,07	100.00			
95%CI	5.34	21.35	0.35	2.46	0.27	2.34	66.99				
	5.62	21.60	0.63	2.74	0.56	2.62	67.15				

Source: NHIFA, 2019.

**Table 3:** Results of correlation analyses.

Type of Care	Mean Age (Years)	Health Insurance Expenditure per Patient (EUR)	Annual Number of Cases per Patient	Mean Age vs. Health Insurance Expenditure per Patient		Mean Age vs. Annual Number of Cases per Patient	
				*r* Value	*p* Value	*r* Value	*p* Value
General practitioner care	38.37	14.29	2.32	−0.007	0.973	−0.007	0.973
Home care	36.41	216.53	1.50	--	--	--	--
Patient transportation	72.79	62.43	3.32	0.511	0.018	0.649	0.002
Ambulatory service	40.55	--	1.00	--	--	0.879	0.000
Outpatient care	37.52	11.83	2.24	−0.030	0.885	−0.453	0.036
Care in care centers	41.00	18.11	1.89	0.945	0.000	0.981	0.000
Laboratory diagnostics	36.58	7.19	1.46	−0.503	0.020	0.306	0.152
CT, MRI	38.17	156.65	1.66	0.178	0.401	−0.285	0.182
PET	0.00	0.00	0.00	--	--	--	--
Acute inpatient care	35.77	502.74	1.02	0.405	0.060	0.117	0.580
Chronic inpatient care	36.70	797.32	1.00	0.973	0.000	0.995	0.000
Disposable instruments, implantations and medicaments falling under itemized accounts	0.00	0.00	0.00	--	--	--	--
Subsidized medicaments	38.77	210.07	2.73	−0.356	0.097	−0.097	0.645
Subsidized medical aids	55.56	66.40	1.45	0.011	0.957	0.237	0.265

Source: own calculations based on data from the NHIFA, 2019.

## Data Availability

Restrictions apply to the availability of these data. Data were obtained from the National Health Insurance Fund Administration (NHIFA) of Hungary and are available from the authors with the permission of the NHIFA.
